# Coupling culturomics and metagenomics sequencing to characterize the gut microbiome of patients with cancer treated with immune checkpoint inhibitors

**DOI:** 10.1186/s13099-025-00694-4

**Published:** 2025-04-11

**Authors:** Khoudia Diop, Babacar Mbaye, Somayeh Nili, Alysé Filin, Myriam Benlaifaoui, Julie Malo, Anne Sophie Renaud, Wiam Belkaid, Sebastian Hunter, Meriem Messaoudene, Karla A. Lee, Arielle Elkrief, Bertrand Routy

**Affiliations:** 1 Centre de Recherche du CHUM, Montreal, Canada; 2 Departement of Hemato-Oncology, Centre Hospitalier de l’Université de Montréal, Montreal, Canada; 3 Departement of Medical Oncology, The Royal Marsden NHS Foundation Trust, London, UK

**Keywords:** Culturomics, Gut microbiome, Metagenomics sequencing, Lung cancer, Melanoma, Immunotherapy

## Abstract

**Background:**

The gut microbiome represents a novel biomarker for melanoma and non-small cell lung cancer (NSCLC) patients treated with immune checkpoint inhibitors (ICI). Gut microbiome metagenomics profiling studies of patients treated with immunotherapy identified bacteria associated with ICI efficacy, while others have been linked to resistance. However, limitations of metagenomics sequencing, such as complex bioinformatic processing requirements, necessity of a threshold for positive detection, and the inability to detect live organisms, have hindered our ability to fully characterize the gut microbiome. Therefore, combining metagenomics with high-throughput culture-based techniques (culturomics) represents an ideal strategy to fully characterize microbiome composition to more robustly position the microbiome as a biomarker of response to ICI.

**Methods:**

We performed culturomics using fecal samples from 22 patients from two academic centres in Canada and the United Kingdom with NSCLC and cutaneous melanoma treated with ICI (cancer group), comparing their microbiome composition to that of 7 healthy volunteers (HV), along with matching shotgun metagenomics sequencing.

**Results:**

For culturomics results, 221 distinct species were isolated. Among these 221 distinct species, 182 were identified in the cancer group and 110 in the HV group. In the HV group, the mean species richness was higher compared to the cancer group (34 vs. 18, respectively, p = 0.002). Beta diversity revealed separate clusters between groups (p = 0.004). *Bifidobacterium spp.* and *Bacteroides spp.* were enriched in HV, while cancer patients showed an overrepresentation of *Enterocloster* species, as well as *Veillonella parvula.* Next, comparing cancer patients’ clinical outcomes to ICI, we observed that among the 20 most abundant bacteria present in non-responder patients, 2 belonged to the genus *Enterocloster,* along with an enrichment of *Hungatella hathewayi and Cutibacterium acnes*. In contrast, responders to ICI exhibited a predominance of *Bacteroides spp*. In NSCLC patients, metagenomics analysis revealed that of the 154 bacteria species isolated through culturomics, 61/154 (39%) were also identified by metagenomics sequencing. Importantly, 94 individual species were uniquely detected by culturomics.

**Conclusion:**

These findings highlight that culturomics and metagenomics can serve as complementary tools to characterize the microbiome in patients with cancer. This integrated approach uncovers specific microbiome signatures that differentiate HV from cancer patients, and identifies specific species associated with therapy response and resistance.

**Supplementary Information:**

The online version contains supplementary material available at 10.1186/s13099-025-00694-4.

## Introduction

The gut microbiome—a community of mostly commensal organisms residing within the human gastrointestinal tract—has rapidly garnered traction in oncology [1]. Preclinical studies first unraveled the unforeseen link between the gut microbiome and response to immune checkpoint inhibitors (ICI) in several malignancies, such as non-small cell lung cancer (NSCLC) and melanoma [2–4]. Pioneering experiments described that ICI activity was abrogated in germ-free mice or after antibiotic administration in specific-pathogen-free mice, pointing to the critical role of the gut microbiome in modulating the antitumor immune response to ICI [2–4]. Antitumor immunity was then restored after fecal microbiota transplantation (FMT) from responder patients or after oral supplementation with specific commensal bacteria associated with response to ICI [2, 4–6]. In parallel, shotgun metagenomics sequencing of patients with NSCLC, melanoma, and renal cell carcinoma demonstrated enrichment of beneficial commensal bacteria in responder patients compared to enrichment of deleterious bacteria in non-responder patients, mirroring observations in preclinical models [2, 7]. Most recently, large and robust meta-analyses in 46,000 patients confirmed the deleterious relationship between pre-treatment antibiotic exposure on survival to ICI [8]. These findings have driven the development of clinical trials aimed at modifying the gut microbiome through various methods, including FMT [9–11]. These early clinical trials have shown that microbiome interventions can circumvent secondary resistance to ICI and potentially to prevent the development of primary resistance to ICI.

Indeed, with more than 50% of cancer patients treated with ICI at some point in their cancer care trajectory, and the majority developing resistance to these therapies, there is an unmet medical need to improve the efficacy of these agents [12, 13]. Moreover, currently available biomarkers to predict response and toxicity to these therapies are neither sensitive nor specific [14]. Given the rapidly evolving field of onco-microbiome research, the tremendous promise of gut microbiome biomarkers, and interventions to enhance immunotherapy efficacy, there have been significant efforts to deeply characterize the gut microbiome of patients undergoing cancer ICI to (1) predict response to these drugs, (2) select patients for appropriate microbiome interventions, and (3) identify key bacterial consortia for development of the next generation of microbiome therapeutics. While shotgun metagenomics sequencing has allowed for deep profiling and characterization of these bacterial communities, sequencing techniques are limited by requirement for advanced bioinformatics support. In addition, shotgun metagenomics sequencing cannot evaluate for organism viability [15]. Lastly, a significant proportion of bacterial hits detected from shotgun metagenomics sequencing are represented by as-of-yet uncharacterised species or strains [15]. Therefore, complementary techniques to counter these limitations are of great interest to propel the field of the gut microbiome forward to improve patient selection, stratification, and outcomes for microbiome interventions.

Culturomics has emerged as a complement to shotgun metagenomics sequencing due to simple workflow, ability to detect live bacteria, to characterize previously unknown species and strains, and to detect taxa at low abundance [16–19]. Despite these advantages, the literature on culturomics in patients treated with ICI remains limited. Specifically, parallel metagenomics sequencing and culturomics describing the overlapping and distinct bacteria detected in patients with cancer has not been reported. To address this, we deeply characterized the gut microbiome using culturomics of 22 patients with advanced melanoma and NSCLC treated with ICI and compared their composition with 7 healthy individuals.

## Materials and methods

### Patients and samples

Human samples were collected from 3 different biobanks, including the CRCHUM NSCLC ethical number CE17.035, CRCHUM healthy volunteers’ biobank ethical number CE20.300 and from the PRIMM study ethical number NCT03643289. Samples were collected by the patients in their homes according to International Human Microbiota Standard (IHMS), and stored in the fridge for 1 day, then brought to the research centre, and immediately at − 80 °C. Responder status was defined by either a partial or complete response to ICI as assessed by the investigator. Patients were considered non-responders if they had stable or progressive disease as assessed by the investigator.

### Bacterial isolation by culturomics

Microbial culturomics was used to explore the bacterial diversity of stool samples and identification was facilitated using the MALDI-TOF MS. For the culture, we used two steps: Firstly, a direct inoculation of the stool sample was performed with 0.3 g of stool resuspended in 1 mL of 1 × PBS. Ten serial dilutions of this suspension were performed, and 50 µL of each dilution was spread on 5% Columbia agar (COS) (Nepean, Ontario, Canada) enriched with sheep blood (ThermoFisher, Montreal Canada), plates were incubated under aerobic and anaerobic conditions using Zip bag (Becton Dickson Mississauga, Ontario, Canada) containing an anaerobic generator, GasPak (Becton Dickson Mississauga, Ontario, Canada) atmospheres at 37 °C for 48 h. Secondly, enrichments were made by adding 200 µL of each sample in liquid broths BACTEC™ vials (Becton Dickson Mississauga, Ontario, Canada) supplemented with 5% of defibrinated sheep blood and 5% of 0.22 µm filtered rumen fluid under both aerobic and anaerobic conditions. For anaerobic conditions, serial dilutions were prepared from the anaerobic BACTEC™ vials and then spread on COS agar at different time points (24 h, day 3, day 7, day 10, day 15, day 21 and day 30) over a period of one month, at 37 °C, using Zip and GasPak generators. For aerobic conditions, serial dilutions were also prepared from aerobic BACTEC™ vials and inoculated on COS agar at the same time points, but under an aerobic atmosphere. The colonies obtained were subcultured after incubation for 48 h at 37 °C, and the purified colonies were identified using MALDI-TOF mass spectrometry. For each colony, a double deposit was made on a 96 MSP microplate and then coated with 2 μL of matrix solution, prepared from saturated α-cyano-4-hydroxycinnamic acid powder mixed with 50% acetonitrile and 2.5% trifluoroacetic acid. The spectra of each colony were then measured using the MicroFlex LT/SH spectrometer and automatically recorded using FlexControl v.3.4 and MALDI Biotyper Compass v4 software for assay preparation and biotyping. The spectra obtained were compared with the MBT Compass BDAL library (Bruker) and our local database. Colonies with a score > 1.9 were identified to the species level. Colonies not identified by MALDI-TOF MS (score < 1.9) were subjected to genomic sequencing for identification.

### *Metagenomics sequencing and analysis*

gDNA was quantified using the Quant-iT™ PicoGreen® dsDNA Assay Kit (Life Technologies). Libraries were generated from 50 ng of gDNA using the NEBNext Ultra II DNA Library Prep Kit for Illumina (New England BioLabs) as per the manufacturer’s recommendations. Adapters and PCR primers were purchased from IDT. Size selection of libraries containing the desired insert size was performed using SparQ beads (Qiagen). Libraries were quantified using the Kapa Illumina GA with Revised Primers-SYBR Fast Universal kit (Kapa Biosystems). Average size fragment was determined using a LabChip GXII (PerkinElmer) instrument. Libraries were normalized and pooled, then denatured in 0.05 N NaOH and neutralized using HT1 buffer. The pool was loaded at 225 pM on an Illumina NovaSeq S4 lane using Xp protocol as per the manufacturer’s recommendations. The run was performed for 2 × 150 cycles (paired-end mode). A phiX library was used as a control and mixed with libraries at 1% level. Base calling was performed with RTA v3.4.4. Program bcl2fastq2 v2.20 was then used to demultiplex samples and generate fastq reads. Shotgun metagenomic sequencing was performed at a read depth of 15 Gb/sample. FASTQ files were processed using the MetaPhlAn4 pipeline as previously described [11].

### Statistical analysis

#### Statistical analysis—culturomics

The detection frequency difference of each species was calculated to compare the microbiota profile obtained by culturomics based on its presence/absence in each sample. Once the frequency of each species in each group was determined, the difference was calculated to determine which species are enriched in each group. The bilateral Chi-squared test with False Discovery Rate (FDR) using the Benjamini–Hochberg method was used to compare frequency differences. Additionally, we conducted an overlap analysis, which compared the species isolated from each group to identify those common to all groups, as well as species specific to each. To compare the differences in numbers of species between different groups, we assessed the normality of the data using the Shapiro–Wilk test using GraphPad Prism version 9 for Windows (GraphPad Software, San Diego, CA, USA, www.graphpad.com). To compare two groups, we used Student's t-test when the data followed a normal distribution. Otherwise, in the presence of non-normal data, we opted for the non-parametric Mann–Whitney test. For multiple comparisons, we used ANOVA followed by Tukey’s test with normal data or the Kruskal–Wallis test followed by Dunn's test with non-normal data. R software (R version 4.4.0) was used to perform principal coordinate analysis (PCoA) with Bray–Curtis dissimilarity to examine the structure and distribution of microbial communities between samples. In addition, a permutation multivariate analysis of variance (PERMANOVA) was used to compare the beta diversity between groups. For multiple comparisons, pairwise PERMANOVA test FDR using the Benjamini–Hochberg test was used to evaluate the difference between groups.

To assess species more frequent across groups (Cancer vs HV and Rvs NR), the Heatmap function in the ComplexHeatmap package was used to visualize data. For data with a normal distribution, a t-test was applied; otherwise, a Wilcoxon test was used. Based on the results of these tests, a vector of p-values was generated. We then filtered the data, retaining only those species whose p-value was less than 0.05. For visualization, Heatmaps were constructed using frequency matrices and annotations for rows (groups) and columns (species), grouped based on the filtered data.

### Statistical analysis—metagenomics

To allow comparison with the culturomics results, we performed frequency comparison within each group, which considers only the presence or absence of each species. For diversity analyses and Linear Discriminant Analysis (LDA), we used the MicrobiomeAnalyst pipeline using the default parameters (https://www.microbiomeanalyst.ca/), which relies on relative abundance or the number of read for each species. Alpha and beta diversity were also assessed using this last pipeline, with a p-value ≤ 0.05 considered statistically significant.

## Results

### Patient characteristics

Twenty-two patients with cancer were included in the study and compared with a control group of seven healthy volunteers (HV). In the cancer group, 13 patients had NSCLC, and 9 had cutaneous melanoma, with a median age of 66 and 61 respectively, and 69% of NSCLC and 67% of melanoma were male (Table 1). All patients with cancer in both cohorts had advanced disease. For the NSCLC cohort, 85% patients had adenocarcinoma with programmed death-ligand 1(PD-L1) tumor proportion score (TPS), a standard prognostic biomarker for patients with NSCLC) [20]. Most patients were treated with single-agent anti- programmed death-1 (PD-1). For the melanoma cohort, 78% of patients had a diagnosis of cutaneous melanoma with *BRAF* wild type status (*BRAF* is the most commonly altered genetic alteration in melanoma and used for treatment decisions), and most patients were treated with combination anti-PD-1 and anti-CTLA-4. In the HV group, the median age was 37, with 43% being male.

**Table 1:** Baseline characteristics of patients and healthy volunteers

NSCLC	N = 11
Age	
Median	64 (52–73)
Sex	
Male	9 (69%)
ECOG performance-status score	
0	8 (61%)
1	4 (31%)
2	1 (8%)
Histology	
Adenocarcinoma	9 (69%)
Squamous	4 (31%)
PDL-1 expresssion	
> 50%	11 (85%)
≤ 50%	2 (15%)
Treatment	
Anti-PD-L1	10 (77%)
Anti-PD-L1 + chemo	2 (15%)
Anti-PD-L1 + Anti CTLA-4 + chemo	1 (8%)

Melanoma	N = 9
Age	
Median	61 (45–81)
Sex	
Male	6 (67%)
ECOG performance-status score	
0	5 (56%)
1	3 (33%)
2	1 (11%)
Cancer Type	
Cutaneous	8 (89%)
Uveal	1 (11%)
BRAF	
Wild Type	7 (78%)
Mutant	1 (11%)
Unknown	1 (11%)
Treatement	
Anti-PD-L1	3 (33%)
Anti-PD-L1 + Anti CTLA-4	6 (67%)

Healthy volunteers (HV)	N=7
Age	
Median	37 (27–42)
Sex	
Male	3 (43%)

### Culturomics profiling reveals higher species richness in healthy volunteers compared to patients with cancer and distinct bacteria associated with cancer

Through culturomics analysis of 29 samples, we identified 221 different species from 102 genera with *Bacteroides*, *Enterococcus*, *Streptococcus* and *Alistipes* being the most common genera present, including three novel species; *Alistipes montrealensis* sp.nov, *Tractidigestivibacter montrealensis* sp. nov and *Gabonibacter chumenis* sp.nov [18, 19, 21] (Table S1).

**Fig. 1:**
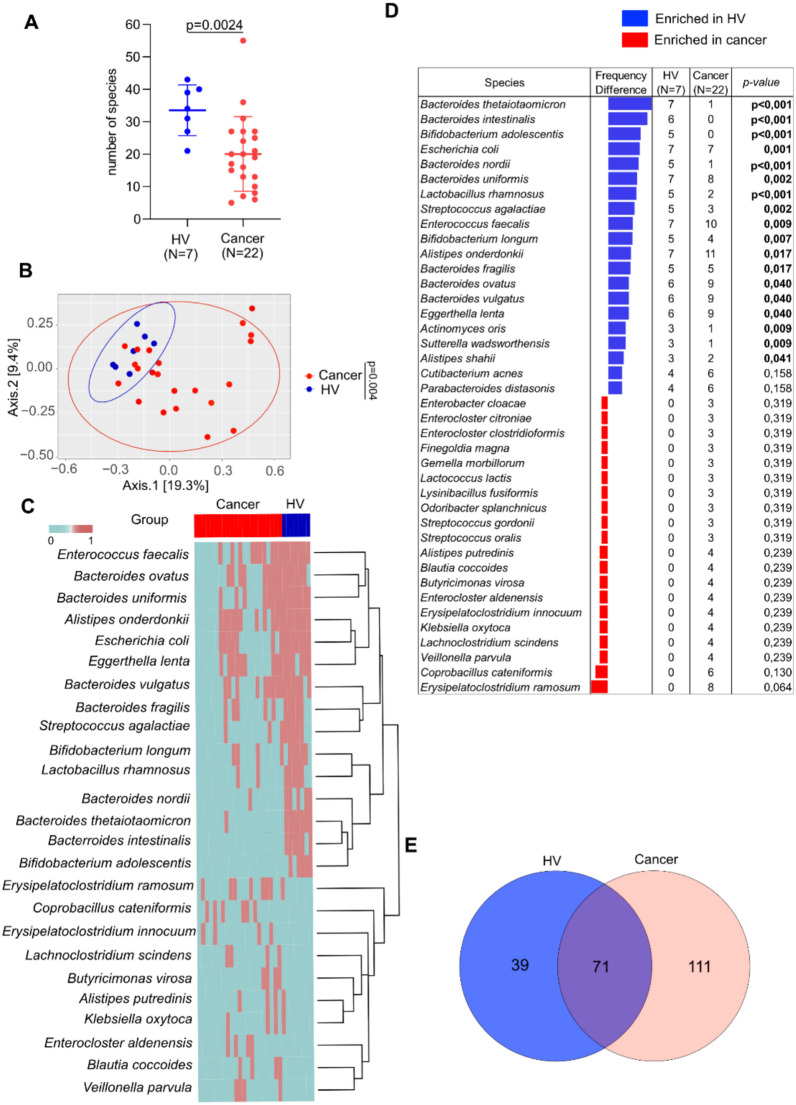
Loss of bacterial diversity in cancer patients compared to HV using culturomics. **A** Number of species isolated in HV and cancer patients (melanoma and NSCLC) (Bilateral Mann–Whitney test). **B** Beta diversity between cancer patients and HV (PERMANOVA test). **C** Heatmap representation between cancer patients and HV. **D** Top 20 species enriched in each group, with bold values indicating significantly enriched results by bilateral Chi-squared test p ≤ 0.05. HV: Healthy volunteers. **E** Venn diagram representation between HV and cancer patients. Cancer- include patients with melanoma and NSCLC

We first compared diversity metrics between the cancer and HV groups. The HV group had significantly more isolated species compared to the cancer group, with 110 different species (mean of 34, range 21–43) compared to 182 different species (mean of 20, range 5–55) (p = 0.0024), respectively, with the exception of one outlier in the cancer group (Fig. 1A**, **Table 2). Additionally, differences were observed between cancer histologies with NSCLC harboring more isolated bacteria than melanoma, with 145 different species (mean of 26, range 16–55) versus 75 different species (mean of 11, range 5–24) (adj p = 0.0067), respectively (Figure S1A, Table S1). Beta diversity analysis also showed clustering of HV compared to cancer patients and differences between HV and distinct cancer histologies (Fig. 1B, Figure S1B), (p = 0.004, p-adj = 0.003 and p-adj = 0.003, respectively).

**Table 2:**
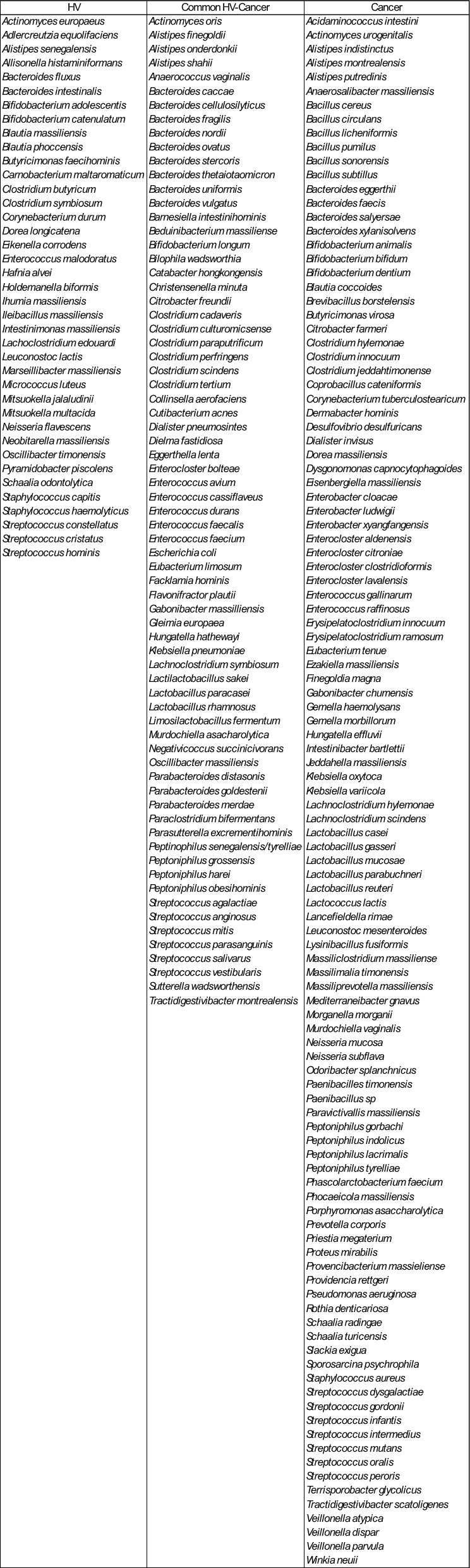
List of bacteria identified with culturomics in healthy volonteers (HV) feces and cancer patients (melanoma and NSCLC)

Next, we performed heatmap representation to determine significant bacteria enriched in both groups. Several species were significantly enriched in the HV group compared to the cancer group including members of the *Bifidobacterium* genus (*B. longum, and B. adolescentis*), *Bacteroides* genus (*B.ovatus*, *B.uniformis*, *B.nordii*, *B.vulgatus*, *B.fragilis* and *B. thetaiotaomicron*) as well as *Alistipes onderdonkii* (Fig. 1C). Conversely, in the cancer group, *Coprobacillus cateniformis*, *Klebsiella oxytoca* and *Veillonella parvula* were more abundant (Fig. 1C). Subsequently, a separate analysis of the top 20 bacteria revealed that 18 species were significantly enriched in HV group. Frequency difference analysis confirmed the enrichment of *B. longum*, *B. adolescentis* and *Bacteroides thetaiotaomicron* in the HV group (Fig. 1D). In addition, using adjusted p-value 14 bacteria remained significantly increased in the HV group (Figure S1C). In the cancer group, we observed a trend for enrichment of two *Enterocloster spp.* (*aldenensis, clostridioformis*), *Veillonella parvula* as well as *Alisitpes putredinis*, *Odoribacter splanchnicus* and *Erysipelatoclostridium ramosum* reclassified as *Thomasclavelia ramosa*. For further characterization, we performed an overlap analysis and found that 71 bacteria were common to both groups, while 39 were unique to HV and 111 were exclusively isolated from cancer patients (Fig. 1E, Table 2). There were several genera exclusive to cancer including: *Enterocloster, Peptoniphilus*, *Bacillus*, *Erysipelatoclostridium*, and *Veillonella*. In the same way*, Eikenella, Oscillibacter* and *Bifidobacterium* genera were only found in HV (Table 2). Altogether these results demonstrate an enrichment in the number as well as specific bacteria previously associated with a healthy gut microbiome in HV, and the presence of specific genera exclusively present in cancer.

### Culturomics profiling reveals distinct species enriched in responders compared to non-responders to immune checkpoint inhibitors

**Fig. 2:**
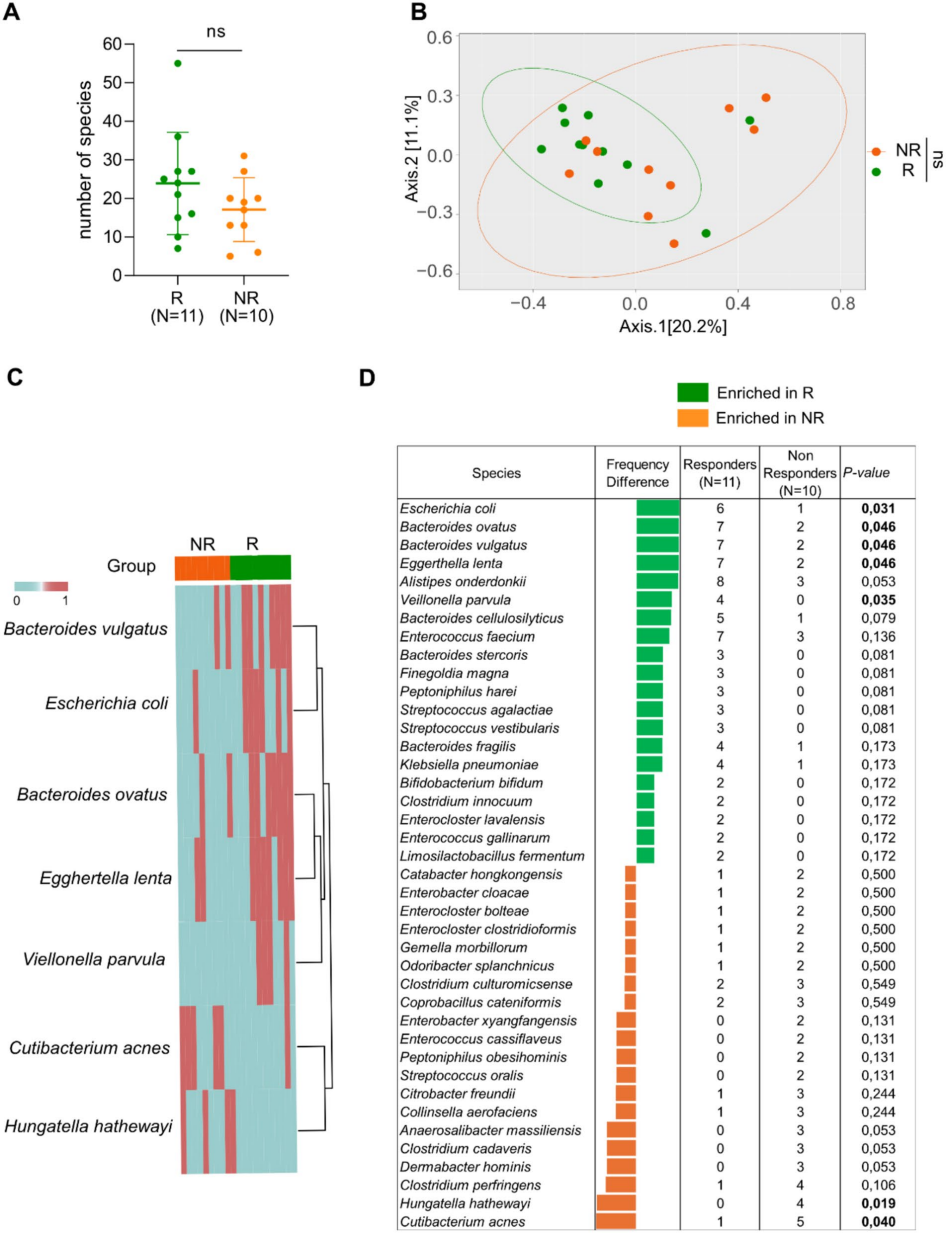
Different Bacterial composition between Responders (R) and Non-responders (NR) cancer patients (melanoma and NSCLC) treated with ICI using culturomics. **A** Number of species isolated in each group (Bilateral Mann–Whitney test). **B** Beta diversity between R vs NR patients (PERMANOVA test). **C.** Heatmap representation between Responders and Non-Responders. **D** Top 20 species enriched in each group, with bold values indicating significantly enriched results by bilateral Chi-squared test p ≤ 0.05. R: Responder; NR: Non-responder

To identify cultivated bacteria associated with responder (R, n = 11) and non-responder (NR, n = 10) status of cancer patients (melanoma and NSCLC) based on objective response rate (ORR) to ICI, we first examined diversity metrics between these two groups. No significant differences were observed in the number of isolated species or in beta diversity (p = 0.17, and p = 0.12, respectively) (Fig. 2A, B). However, *Hungatella hathewayi* and *Cutibacterium acnes* were significantly enriched in NR. Conversely *Bacteroides ovatus, B. vulgatus, Escherichia coli*, *Eggerthella lenta* and *Veillonella parvula* were significantly increased in R patients (Fig. 2C). Subsequently, addressing the 20 most frequent isolated bacteria in each group, NR patients exhibited a total of two *Enterocloster* species (*E.bolteae* and *E.clostridioformis*), three *Clostridium* species (*C.culturomicsense, C.cadaveris* and *C.perfringens*) and *Hungatella hathewayi* and *Cutibacterium acnes* were the only significantly increased bacteria in this group (Fig. 2D). Conversely, the following species were significantly enriched in the R group with the predominance of *Bacteroides* genus (*ovatus*, *vulgatus*, *cellulosilyticus*, *stercoris,* and *fragilis,*) and as well as *Escherichia coli*, *Eggerthella lenta,* and *Veillonella parvula* were also enriched in this group (Fig. 2D). Of note, using Benjamini–Hochberg method to calculate the adjusted p-value, there no longer a significant difference between both groups (Figure S2A).

Next, we performed the same analysis in the subgroup of NSCLC patients (n = 13), and identified similar findings as for the pooled NSCLC and melanoma results. NR (n = 6) exhibited an enrichment of three *Enterocloster spp*. (*aldenensis, bolteae* and *clostridioformis*) as well as *Hungatella hatewayi* and *Clostridium perfringens* (Figure S2B&C*)*. In contrast, the R (n = 7) group showed an enrichment of *Bacteroides* genus (Figure S2B). These findings align with previous studies that differentiate the gut microbiomes of R from NR, further reinforcing the role of the gut microbiome in segregating patient phenotypes to ICI.

### Overlap analysis between culturomics and metagenomics reveal the complementary profiling techniques

**Fig. 3:**
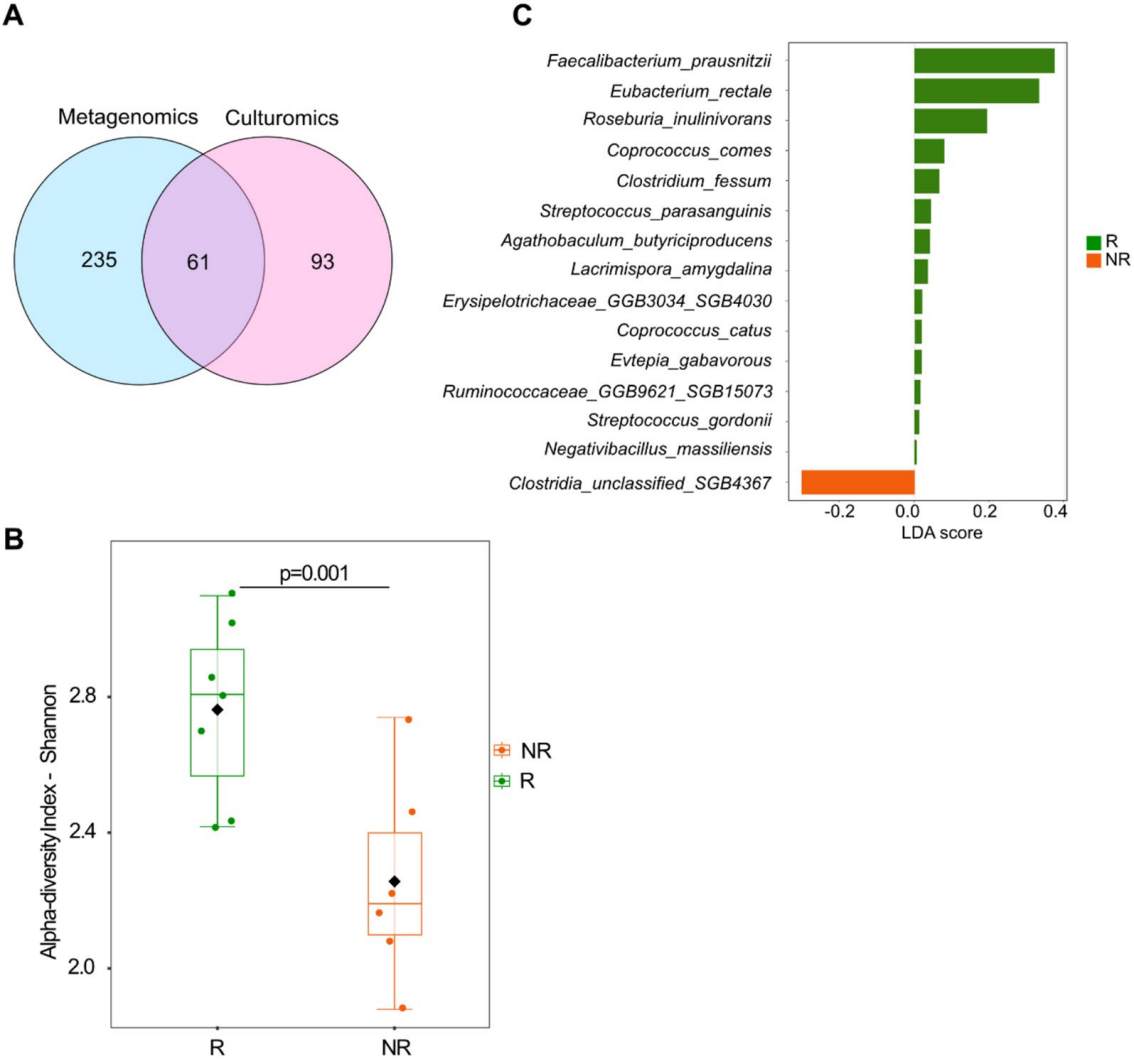
Culturomics and metagenomics represent complementary methods to characterize gut microbiome of NSCLC patients. **A** Venn diagram representation comparing culturomics and metagenomics results of patients with NSCLC; the number of species shared and distinct between both methods are represented in the circle. **B** Alpha (α) diversity Shannon index comparing 7R and 6NR patients with NSCLC by metagenomics. **C** Linear discriminant analysis (LDA) on metagenomics sequencing results comparing R and NR; logarithmic LDA score > 0 indicating a higher relative abundance in R compared to NR

To understand the commonalities and differences between culturomics results and metagenomics, we performed shotgun metagenomics in all NSCLC patients. Metagenomics sequencing detected a higher number of species with 721 taxa (mean of 164 range, 32–253) compared to 154 different species (mean of 26, range 16–55) (p-value < 0.001) identified by culturomics (Tables S2-S3). Among the 721 taxa, 425 were unassigned at the species level (Table S2). Of the 296 species identified (assigned at the species level) by metagenomics (mean of 85, range 24–122), 61 species were common with culturomics, while 93 species were found only by culturomics (p-value < 0.001) (Fig. 3A, Tables S3-S4).

We then analyzed the metagenomics data taking into consideration the patient clinical outcome. As previously published in the literature [2, 3], alpha diversity was significantly higher in R compared to NR (p = 0.001) (Fig. 3B), with no difference in beta diversity (p = 0.27) (Figure S3A). Linear discriminant analysis (LDA) between R and NR NSCLC revealed that *Faecalibacterium prausnitzii, Eubacterium rectale, Roseburia inulinivorans, Coprococcus comes,* and *Agathobaculum butyriciproducens* species were enriched with R status (Fig. 3C). Conversely, only *Clostridia_unclassified_SGB4367* species was increased in NR NSCLC patients (Fig. 3C). In addition, we performed frequency comparisons between R and NR groups which revealed only for non-adjusted p-value additional enriched species, including *Lacrimispora amygdalina*, *Evtepia gabavorous*, *Streptococcus parasanguinis* and *Agathobaculum butyriciproducens* in R, and *Clostridia-unclassified-SGB4367*, *Ruminococcaceae-GGB58485-SGB80143*, *Enterococcaceae-GGB33516-SGB5434* and *Anaerofilum-sp-BX8* in NR (Figure S3B and C).

These results reinforce previous findings demonstrating higher alpha diversity as well as specific bacteria associated with response to ICI in NSCLC such as *Faecalibacterium* and *Roseburia* and also highlight the complementary nature of these two orthogonal gut microbiome profiling techniques.

## Discussion

The gut microbiome has emerged as one of the most promising biomarkers for predicting clinical outcomes in patients treated with ICI [22, 23]. Despite this, studies in patients treated with ICI combining shotgun metagenomics sequencing and culturomics are lacking. This study performed dual profiling in 22 advanced NSCLC and melanoma patients, focusing on high-throughput culturomics combined with metagenomics sequencing for the NSCLC patients. Our study supports the concept of cancer-related dysbiosis, characterized by lower bacterial counts and altered microbiome composition in patients with cancer compared to healthy individuals. In addition, we found this dysbiosis to be defined by bacteria associated with cancer development, such as *Enterocloster* species and *Veillonella* species [23, 24]. Culturomics further validated the presence of *Hungatella hathewayi* and *Streptococcus spp*. in non-responders, while *Bacteroides spp.* were prevalent in responders. Importantly, our study found a significant overlap between sequencing and culture-based techniques but also identified distinct bacteria using culturomics, highlighting its utility to detect low-abundance species that might be filtered out by thresholds required for shotgun metagenomics sequencing.

Yonekura et al. investigated in preclinical models, how cancer can cause stress-induced ileopathy through β-adrenergic receptor activation [23]. This ‘stress ileopathy’, associated with sustained *Clostridium spp*.-related dysbiosis. While numerous cohort studies have profiled the gut microbiome of cancer vs. healthy controls, these studies do not provide any causal links and reinforce the open ‘chicken or egg’ conundrum of the cancer microbiome. For example, in a large cohort study by Gao et al., which included 156 colorectal cancer patients and 104 healthy controls, the authors observed a significant reduction in microbial diversity [25]. These cohort studies, among others [26], emphasize the consistent patterns of microbial dysbiosis fingerprints across various cancer types compared to healthy controls, suggesting that microbial shifts could potentially serve as a diagnostic biomarker. This is corroborated by our study, which also found significantly reduced bacterial diversity in cancer patients compared to healthy controls.

Several studies have utilized culturomics to study gut microbiome differences in various health conditions, but its application in distinguishing between cancer and healthy gut microbiomes is still emerging. To the best of our knowledge, our paper is one of the first to compare cancer-associated and healthy microbiomes using both culturomics and metagenomics techniques. For example, Dubourg et al*.* used culturomics to study the gut microbiota composition in healthy individuals, and identified specific bacteria not detected by metagenomics sequencing which led to the addition of 531 species to the human gut repertoire [27]. Importantly, our comparative analysis between culturomics with the gold-standard of metagenomics sequencing found a significant overlap in species identified by the two techniques, with metagenomics identifying a higher proportion of species overall, as expected. However, 94 species were uniquely identified using culturomics. Interestingly, metagenomics was able to identify key bacteria associated with ICI efficacy such as *Feacalibaterium prausnitzii* [27] which is a fastidious bacterium typically difficult to culture. In addition, our culturomics findings re-capitulated prior findings of cancer vs. healthy microbiome imprint, such as enrichment of *Bifidobacterium* genus including *B. longum, and B. adolescentis*, *Bacteroides* species as well as *Alistipes onderdonkii* in healthy individuals [28, 29]. The present findings have important clinical implications in distinguishing health individuals from those with disease using gut microbiome profiling and in better identifying gut microbiome differences between R and NR to ICI. Notwithstanding the increasing evidence pointing to the modulation of the response to ICI by the gut microbiome, the mechanism remains elusive. Immunosuppressive bacteria such as *Enterocloster* or other *Clostridia* species can modulate host immune responses by producing immunosuppressive metabolites, which may suppress T-cell activation and promote regulatory T-cell differentiation [30]. Additionally, these bacteria influence the expression of mucosal addressin cell adhesion molecule-1 (MAdCAM-1), facilitating the recruitment of regulatory T cells to the gut-associated lymphoid tissue and contributing to a tolerogenic microenvironment. This immune modulation can dampen anti-tumor immune responses and reduce the efficacy of ICI [30].

Despite this being the first study to concurrently evaluate the microbiome of healthy individuals vs. cancer patients and to examine the gut microbiome composition of responders to ICI and non-responders, our study is limited by small sample size which reduces the statistical power to obtain significant adjusted p-values, and lack of functional validation. One limitation of our study is the use of frozen rather than fresh samples. Thawed samples exhibit reduced bacterial viability, particularly affecting anaerobes, which may have influenced our culturomics results. In addition, the healthy volunteer control group was significantly younger than the cancer group, pointing to the known impact of age on bacterial diversity [31]. Future studies with larger sample sizes should therefore account for participant demographics such as age and sex. Despite these limitations, our study reinforces the need to complement metagenomics sequencing with culturomics in future microbiome studies.

## Conclusion

This present study highlights the addition of culturomics to metagenomics, demonstrating key differences in alpha diversity and species-level composition between healthy volunteers and cancer patients, as well as between responders vs. non-responders to ICI. It also reinforces the importance of using complementary techniques to more robustly characterize the cancer-associated microbiome for future applications in cancer detection and biomarker discovery.

## Supplementary Information

Supplementary Material 1.

Supplementary Material 2.

## Data Availability

No datasets were generated or analysed during the current study.

## Abbreviations

ICI: Immune checkpoint inhibitors
HV: Healthy volunteers
NSCLC: Non-small cell lung cancer

## References

[1] Hanahan D. Hallmarks of cancer: new dimensions. Cancer Discov. 2022;12(1):31–46. 35022204 10.1158/2159-8290.CD-21-1059

[2] Routy B, Chatelier EL, Derosa L, Duong CPM, Alou MT, Daillère R, et al. Gut microbiome influences efficacy of PD-1–based immunotherapy against epithelial tumors. Science. 2018;359(6371):91–7. 29097494 10.1126/science.aan3706

[3] Gopalakrishnan V, Spencer CN, Nezi L, Reuben A, Andrews MC, Karpinets TV, et al. Gut microbiome modulates response to anti-PD-1 immunotherapy in melanoma patients. Science. 2018;359(6371):97–103. 29097493 10.1126/science.aan4236 PMC5827966

[4] Matson V, Fessler J, Bao R, Chongsuwat T, Zha Y, Alegre ML, et al. The commensal microbiome is associated with anti-PD-1 efficacy in metastatic melanoma patients. Science. 2018;359(6371):104–8. 29302014 10.1126/science.aao3290 PMC6707353

[5] Messaoudene M, Pidgeon R, Richard C, Ponce M, Diop K, Benlaifaoui M, et al. A Natural Polyphenol Exerts Antitumor Activity and Circumvents Anti-PD-1 Resistance through Effects on the Gut Microbiota. Cancer Discov. 2022. 10.1158/2159-8290.CD-21-0808. 35031549 10.1158/2159-8290.CD-21-0808 PMC9394387

[6] Derosa L, Routy B, Fidelle M, Iebba V, Alla L, Pasolli E, et al. Gut Bacteria Composition Drives Primary Resistance to Cancer Immunotherapy in Renal Cell Carcinoma Patients. Eur Urol août. 2020;78(2):195–206. 10.1016/j.eururo.2020.04.044 32376136

[7] Derosa L, Hellmann MD, Spaziano M, Halpenny D, Fidelle M, Rizvi H, et al. Negative association of antibiotics on clinical activity of immune checkpoint inhibitors in patients with advanced renal cell and non-small-cell lung cancer. Ann Oncol Off J Eur Soc Med Oncol. 1 juin 2018;29(6):1437‑44. 10.1093/annonc/mdy103 PMC6354674 29617710

[8] A E, Eo MS, J M, Cm V, P G, A D, et al. Antibiotics are associated with worse outcomes in lung cancer patients treated with chemotherapy and immunotherapy. NPJ Precis Oncol [Internet]. 16 juill 2024 [cité 10 oct 2024];8(1). Disponible sur: https://pubmed.ncbi.nlm.nih.gov/39014160/10.1038/s41698-024-00630-w PMC11252311 39014160

[9] D D, Ak D, Ja M, Rr R, Jm C, Rm M, et al. Fecal microbiota transplant overcomes resistance to anti-PD-1 therapy in melanoma patients. Science [Internet]. 2 mai 2021 [cité 10 oct 2024];371(6529). Disponible sur: https://pubmed.ncbi.nlm.nih.gov/33542131/10.1126/science.abf3363 PMC8097968 33542131

[10] Baruch EN, Youngster I, Ben-Betzalel G, Ortenberg R, Lahat A, Katz L, et al. Fecal microbiota transplant promotes response in immunotherapy-refractory melanoma patients. Science. 5 févr 2021;371(6529):602‑9. 10.1126/science.abb5920 33303685

[11] Routy B, Lenehan JG, Miller WH, Jamal R, Messaoudene M, Daisley BA, et al. Fecal microbiota transplantation plus anti-PD-1 immunotherapy in advanced melanoma: a phase I trial. Nat Med août. 2023;29(8):2121–32. 10.1038/s41591-023-02453-x 37414899

[12] Gandhi L, Rodríguez-Abreu D, Gadgeel S, Esteban E, Felip E, De Angelis F, et al. Pembrolizumab plus chemotherapy in metastatic non-small-cell lung cancer. N Engl J Med. 2018;378(22):2078–92. 29658856 10.1056/NEJMoa1801005

[13] Robert C, Long GV, Brady B, Dutriaux C, Di Giacomo AM, Mortier L, et al. Five-year outcomes with nivolumab in patients with wild-type BRAF advanced melanoma. J Clin Oncol. 2020;38(33):3937–46. 32997575 10.1200/JCO.20.00995 PMC7676881

[14] Sharma P, Hu-Lieskovan S, Wargo JA, Ribas A. Primary, adaptive, and acquired resistance to cancer immunotherapy. Cell. 2017;168(4):707–23. 28187290 10.1016/j.cell.2017.01.017 PMC5391692

[15] Thomas AM, Segata N. Multiple levels of the unknown in microbiome research. BMC Biol. 2019;17(1):48. 31189463 10.1186/s12915-019-0667-z PMC6560723

[16] Lagier JC, Dubourg G, Million M, Cadoret F, Bilen M, Fenollar F, et al. Culturing the human microbiota and culturomics. Nat Rev Microbiol. 2018;16(9):540–50. 29937540 10.1038/s41579-018-0041-0

[17] Bonnet M, Lagier JC, Raoult D, Khelaifia S. Bacterial culture through selective and non-selective conditions: the evolution of culture media in clinical microbiology. New Microbes New Infect. 2020;34:100622. 31956419 10.1016/j.nmni.2019.100622 PMC6961714

[18] Routy B, Richard C, Benlaïfaoui M, Lapierre SG, Armstrong N, Al-Saleh A, Boko M, Jacq M, Watson IR, Mihalcioiu C, et al. Characterization of Alistipes montrealensis sp. nov., isolated from human feces of a patient with metastatic melanoma treated with immune checkpoint inhibitors. Microbiol Res. 2022;13(1):140–51. 10.3390/microbiolres13010012.

[19] Benlaïfaoui M, Richard C, Diop A, Naimi S, Belkaid W, Bernet E, et al. Tractidigestivibacter montrealensis sp. nov., a new member of human gut microbiota isolated from a healthy volunteer. FEMS Microbiol Lett. 2023;370:fnad058. 37348476 10.1093/femsle/fnad058 PMC10396324

[20] Reck M, Jotte RM, Socinski MA. Atezolizumab treatment of nonsquamous NSCLC. N Engl J Med. 2018;379(12):1188. 30231231 10.1056/NEJMc1809195

[21] Diop K, Pidgeon R, Diop A, Benlaïfaoui M, Belkaid W, Malo J, et al. Characterization and description of Gabonibacter chumensis sp. nov., isolated from feces of a patient with non-small cell lung cancer treated with immunotherapy. Arch Microbiol. 2023;205(10):338. 37742282 10.1007/s00203-023-03671-0 PMC10518271

[22] Routy B, Jackson T, Mählmann L, Baumgartner CK, Blaser M, Byrd A, Corvaia N, Couts K, Davar D, Derosa L, Hang HC, Hospers G, Isaksen M, Kroemer G, Malard F, McCoy KD, Meisel M, Pal S, Ronai Z, Segal E, Zitvogel L. Melanoma and microbiota: current understanding and future directions. Cancer Cell. 2024;42(1):16–34. 10.1016/j.ccell.2023.12.003. 38157864 10.1016/j.ccell.2023.12.003 PMC11096984

[23] Derosa L, Iebba V, Silva CAC, Piccinno G, Wu G, Lordello L, et al. Custom scoring based on ecological topology of gut microbiota associated with cancer immunotherapy outcome. Cell. 2024;187(13):3373-3389.e16. 38906102 10.1016/j.cell.2024.05.029

[24] Yonekura S, Terrisse S, Alves Costa Silva C, Lafarge A, Iebba V, Ferrere G, et al. Cancer induces a stress ileopathy depending on β-adrenergic receptors and promoting dysbiosis that contributes to carcinogenesis. Cancer Discov. 2022;12(4):1128–51. 34930787 10.1158/2159-8290.CD-21-0999

[25] Gao H, Korn JM, Ferretti S, Monahan JE, Wang Y, Singh M, et al. High-throughput screening using patient-derived tumor xenografts to predict clinical trial drug response. Nat Med. 2015;21(11):1318. 26479923 10.1038/nm.3954

[26] Bilen M, Dufour JC, Lagier JC, Cadoret F, Daoud Z, Dubourg G, et al. The contribution of culturomics to the repertoire of isolated human bacterial and archaeal species. Microbiome. 2018;6:94. 29793532 10.1186/s40168-018-0485-5 PMC5966928

[27] Bredon M, Danne C, Pham HP, Ruffié P, Bessede A, Rolhion N, et al. Faecalibaterium prausnitzii strain EXL01 boosts efficacy of immune checkpoint inhibitors. Oncoimmunology. 2024;13(1):2374954. 38957477 10.1080/2162402X.2024.2374954 PMC11218805

[28] Wang T, Cai G, Qiu Y, Fei N, Zhang M, Pang X, et al. Structural segregation of gut microbiota between colorectal cancer patients and healthy volunteers. ISME J févr. 2012;6(2):320–9. 10.1038/ismej.2011.109 PMC3260502 21850056

[29] Haberman Y, Kamer I, Amir A, Goldenberg S, Efroni G, Daniel-Meshulam I, et al. Gut microbial signature in lung cancer patients highlights specific taxa as predictors for durable clinical benefit. Sci Rep. 2023;13(1):2007. 36737654 10.1038/s41598-023-29136-4 PMC9898251

[30] Fidelle M, Rauber C, Alves Costa Silva C, Tian AL, Lahmar I, de La Varende ALM, et al. A microbiota-modulated checkpoint directs immunosuppressive intestinal T cells into cancers. Science. 2023;380(6649):2296. 10.1126/science.abo2296 37289890

[31] Scepanovic P, Hodel F, Mondot S, Partula V, Byrd A, Hammer C, et al. A comprehensive assessment of demographic, environmental, and host genetic associations with gut microbiome diversity in healthy individuals. Microbiome. 2019;7(1):130. 10.1186/s40168-019-0747-x. 31519223 10.1186/s40168-019-0747-x PMC6744716

