# Supplementary material for: Coupling culturomics and metagenomics sequencing to characterize the gut microbiome of patients with cancer treated with immune checkpoint inhibitors
Source: Gut Pathog. 2025 Apr 11;17:21. doi: 10.1186/s13099-025-00694-4 (PMC11992761; doi:10.1186/s13099-025-00694-4)

**Coupling culturomics and metagenomics to characterize the gut microbiome of patients with cancer treated with immune checkpoint inhibitors**

**Authors:** Khoudia Diop, Babacar Mbaye, Somayeh Nili, Alysé Filin, Myriam Benlaifaoui, Julie Malo, Anne Sophie Renaud, Wiam Belkaid, Sebastian Hunter, Meriem Messaoudene, Karla A. Lee, Arielle Elkrief, and Bertrand Routy

**SUPPLEMENTARY DATA**

**Supplementary table 1. Culturomics results**

**See Excel file, sheet TableS1**

**Supplementary Table 2. Metagenomics sequencing results for the NSCLC patients**

**See Excel file, sheet TableS2**

**Supplementary Table 3. Species descriptive results for Metagenomics and culturomics for all samples**

**See Excel file, sheet TableS3**

**Supplementary Table 4. List of bacteria identified with culturomics, with metagenomics and the common between the two technics in NSCLC patients feces**

**See Excel file, sheet TableS4**

**Supplementary Figure 1. Culturomics results between HV vs different cancer patients (melanoma and NSCLC). A**. Number of species isolated in each group; adjusted p-value was calculated by using Dunn's test multiple comparisons **B**. Beta diversity between groups (Pairwise Adonis test with FDR adjusted p-value using Benjamini-Hochberg method). **C**. Top 20 species enriched in HV and Cancer patients patients with bold values indicating significantly enriched results by bilateral Chi-squared test p≤ 0.05.

**
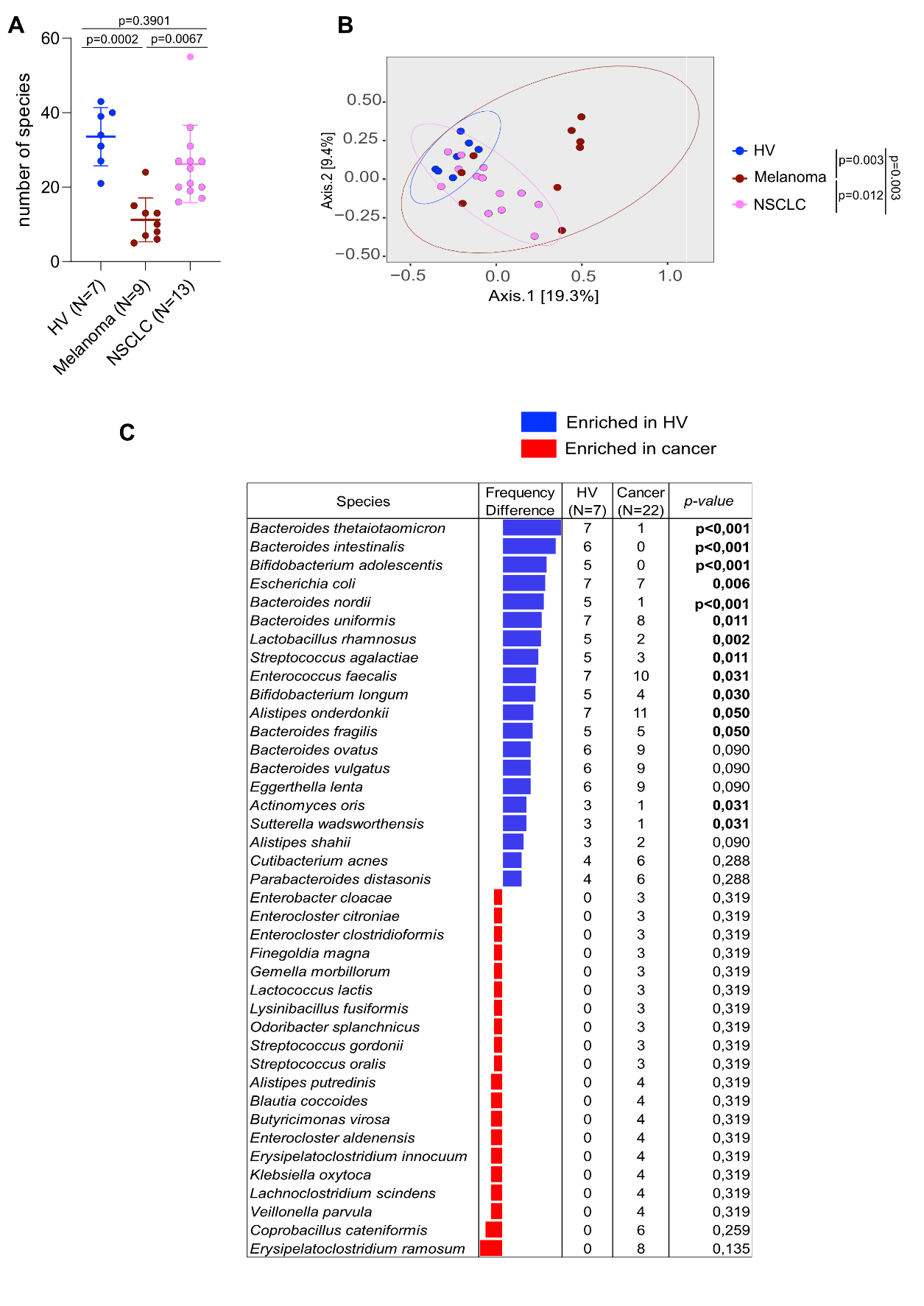
**

**Supplementary Figure 2. Culturomics result between R and NR in cancer patients. A**. Top 20 species enriched in R and and NR patients adjusted p-values were calculated by bilateral Chi-squared test with FDR, Benjamini-Hochberg method, adj p≤0.05. **B**. Top 20 species enriched in each group R and NR in NSCLC patients with bold values indicating significantly enriched results by bilateral Chi-squared test p≤ 0.05. **C**. Top 20 species enriched in each group R and NR in NSCLC patients with bold values indicating significantly enriched results by bilateral Chi-squared with FDR, Benjamini-Hochberg method adj p ≤ 0.05.

**
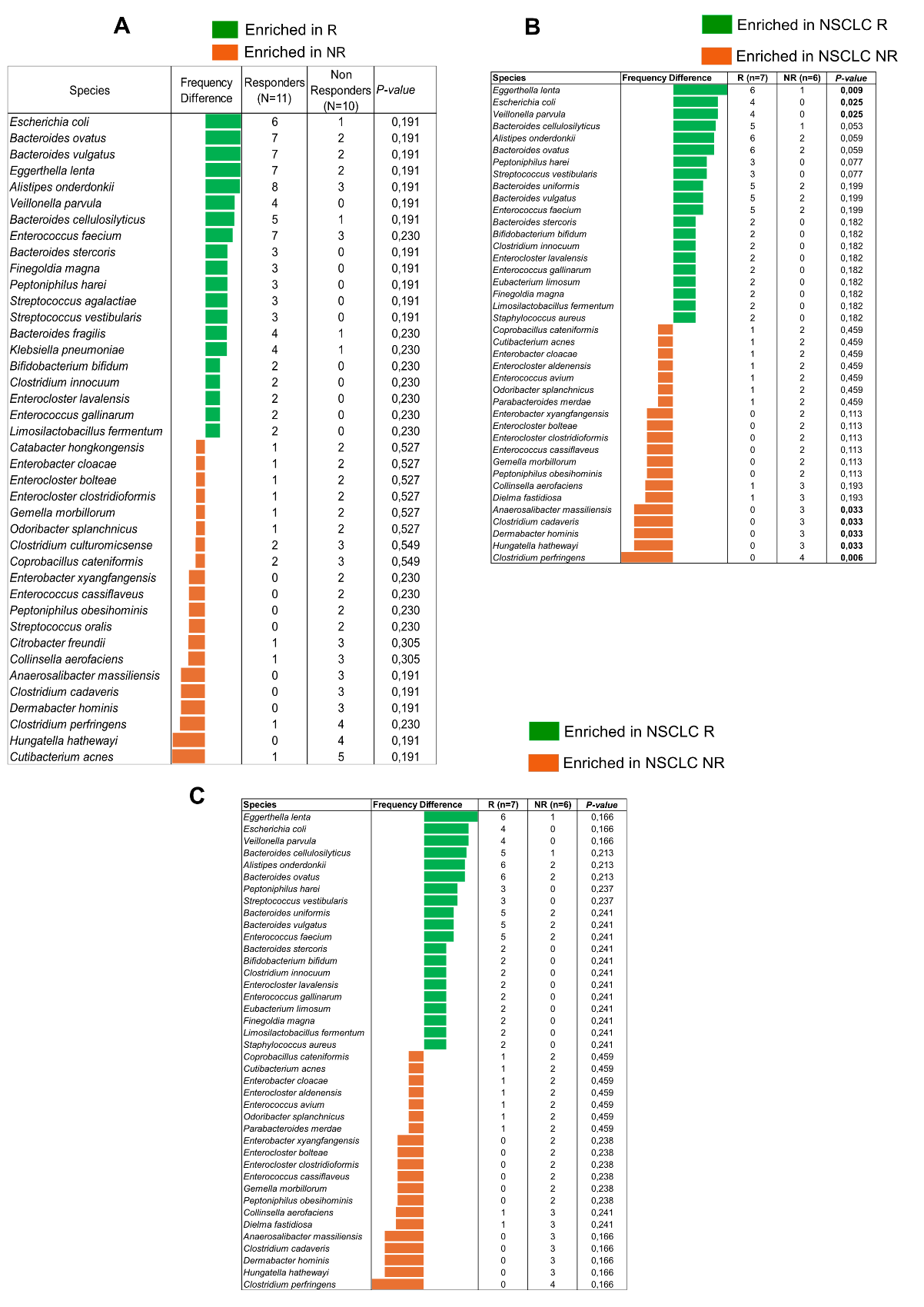
**

**Supplementary Figure 3. Metagenomics results between R and NR in NSCLC patients. A.** Beta diversity between groups. **B**. Top 20 species enriched in each group R and NR p-values were calculated by bilateral Chi-squared test p≤ 0.05. **C**. Top 20 species enriched in each group R and NR p-values were calculated by bilateral Chi-squared with FDR, Benjamini-Hochberg method adj p ≤ 0.05.


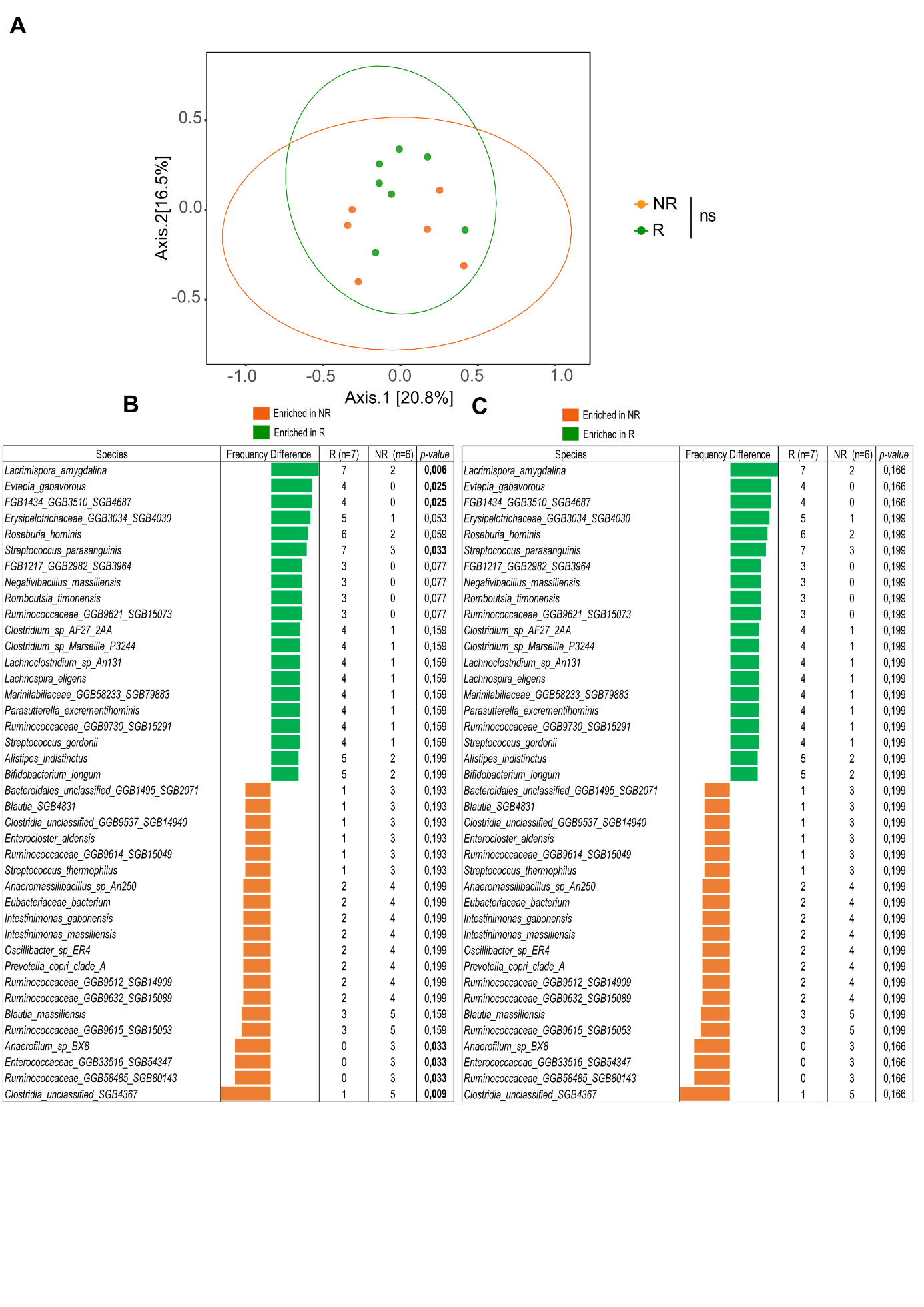

Supplement: Supplementary file 1 — Supplementary Material 1. [file 13099_2025_694_MOESM1_ESM.docx]
